# Multielement Analysis of Selected Superfood Seeds and Grains Using ICP-OES: Sources of Essential and Toxic Elements

**DOI:** 10.3390/molecules31091374

**Published:** 2026-04-22

**Authors:** Elżbieta Maćkiewicz, Piotr Wysocki, Małgorzata Iwona Szynkowska-Jóźwik

**Affiliations:** Institute of General and Ecological Chemistry, Faculty of Chemistry, Lodz University of Technology, Zeromskiego 114, 90-543 Lodz, Poland; piotr.wysocki@p.lodz.pl (P.W.); malgorzata.szynkowska@p.lodz.pl (M.I.S.-J.)

**Keywords:** superfood, elemental analysis, ICP-OES, macroelement, trace element, toxic elements

## Abstract

The term ‘superfoods’ refers to a rapidly expanding group of food products that have gained increasing global interest due to their high nutritional value and association with health-oriented dietary patterns. Many superfoods, particularly grains and seeds, are rich sources of essential minerals, plant protein, dietary fibre, and bioactive compounds, making them valuable components of gluten-free, vegetarian, and vegan diets. The aim of this study was to evaluate the elemental composition of selected superfood grains and seeds and to verify the reliability of manufacturers’ declarations. The analyses confirmed that the investigated samples possess a rich macro- and trace elemental composition, with pronounced differences among product groups. Based on median concentrations, pumpkin and hemp seeds were characterized by generally high levels of Mg, K, P, Fe, Mn, and Zn, whereas chia seeds exhibited notably elevated Ca content. In contrast, quinoa and amaranth showed comparatively lower elemental concentrations. Most of the results obtained for the analysed products are within the permissible deviation from the value declared on the packaging, as specified in the relevant EU regulations. The presence of potentially toxic elements, including Al, Pb, and Cd, was also detected. Cadmium accumulation was of particular concern in flax seeds, where all samples exceeded the limit of quantification and approached permissible levels. Principal component analysis revealed clear clustering patterns, indicating similarities between amaranth and quinoa, as well as between hemp and pumpkin seeds, while chia and flax seeds formed distinct groups. These results highlight both the nutritional potential of superfoods and the necessity for independent verification of their elemental composition.

## 1. Introduction

Recent years have seen a marked increase in the popularity of the concept of “superfoods” among consumers. Superfoods are defined as plant-based products that have been utilised for millennia in diverse regions worldwide as remedies for ailments or as components of the diets of indigenous populations [1,2]. However, it should be noted that the term ‘superfood’ does not equate to ‘functional food’. The latter is defined as “a food can be regarded as ‘functional’ if it is satisfactorily demonstrated to effect beneficially one or more target functions in the body, beyond adequate nutritional effects, in a way which is relevant to either the state of well-being and health or the reduction in the risk of a disease” [3]. Furthermore, it is evident that the contemporary designation “superfoods” is exclusively employed for promotional and marketing objectives within the field, devoid of any scientific substantiation [1].

The distinguishing characteristics of these products are their high nutrient density and elevated levels of essential health components often lacking in the diet, such as omega-3 polyunsaturated fatty acids. The group of so-called superfoods includes both exotic and commonly known products. Increasing consumer awareness of their health-promoting properties has led to a rise in their popularity [4,5,6]. Maintaining a healthy lifestyle—through regular physical activity, avoidance of stimulants, and proper nutrition—supports long-term health. Scientific evidence indicates that regular consumption of small amounts of superfoods can help supplement deficiencies in minerals, healthy fats, protein, and antioxidants. The growing availability of plant-based diets has been associated with benefits for cardiovascular health, type 2 diabetes, and obesity. In addition, environmental concerns, including water conservation and sustainable agriculture, are driving a shift toward plant-based diets, increasing the availability and market share of such products [7,8,9]. Consumer interest in these products is influenced by factors such as nutritional trends, health awareness, the functional properties of superfoods (e.g., antioxidant and anticancer effects), and the appeal of novel flavours. Superfoods include: (i) fruits and fruit products (e.g., goji berry, acai, pomegranate, chokeberry), (ii) nuts and seeds (e.g., chia, flax, hemp), (iii) grains (e.g., amaranth, buckwheat, quinoa, cocoa, oat), (iv) roots and tubers (e.g., ginger, turmeric, ginseng), and (v) vegetables and other products (e.g., kale, spirulina, chlorella, pollen) [10].

Therefore, the research focuses on various aspects using different research techniques, starting from the content of: polyunsaturated fatty acids [11,12,13,14,15,16,17], amino acids [11,12,13,15,17,18,19], polyphenolic compounds [13,14,20,21,22], vitamins and minerals [11,13,14,15,17,18,20,23], and antioxidant capacity and antimicrobial activity [11,13,17,20,21,24,25]. Furthermore, analyses of the content of macroelements such as Ca, Mg, S and P, in addition to toxic elements including heavy metals, are included in the scientific literature [11,14,15,16,20,21,23,26,27,28,29,30]. Another aspect of research is the analysis of adulteration of such products, which is being detected with increasing frequency, especially in relation to more expensive products or spices [31,32,33,34].

This study examined a selection of seeds and grains that have been classified as superfoods. These included amaranth grains, chia seeds, flax seeds, hemp seeds, pumpkin seeds, and quinoa grains. The objective of the present study was to evaluate and contrast the content of macro- and microelements (Ca, Co, Cr, Cu, Fe, K, Mg, Mn, Mo, P, S, Zn), as well as toxic elements (Ag, Al, Ba, Cd, Ni, Pb, Sn, Sr, Ti) in the examined samples. In addition, the obtained results were compared with the values declared by the manufacturers. The analyses were conducted utilising inductively coupled plasma optical emission spectrometry (ICP-OES).

## 2. Results and Discussion

### 2.1. A Comparison of Groups of the Analysed Samples

#### 2.1.1. Toxic Elements

The samples of superfood seeds and grains were divided into groups according to their classification and subjected to statistical analysis. Table 1 presents the results of the analysis of toxic element content for individual groups of grain and seed samples, which have been divided into categories according to type. The minimum, maximum and median values within each group are also indicated.

Conversely, for Ag, an undesirable heavy metal in food, high concentrations were not obtained. A mere four samples yielded results that exceeded the limit of quantification, with a maximum result of 0.317 mg/L observed in sample 38, which was an organically certified pumpkin seed sample.

For another element toxic to humans, Al, concentrations were obtained ranging from below the LOQ (sample 29, certified hemp seeds) to a maximum value of 52.37 mg/L (sample 25, flax seed, uncertified sample). The mean and median values for Al were found to be comparable, suggesting that the majority of the samples analysed exhibited similar Al content.

**Figure 1:**
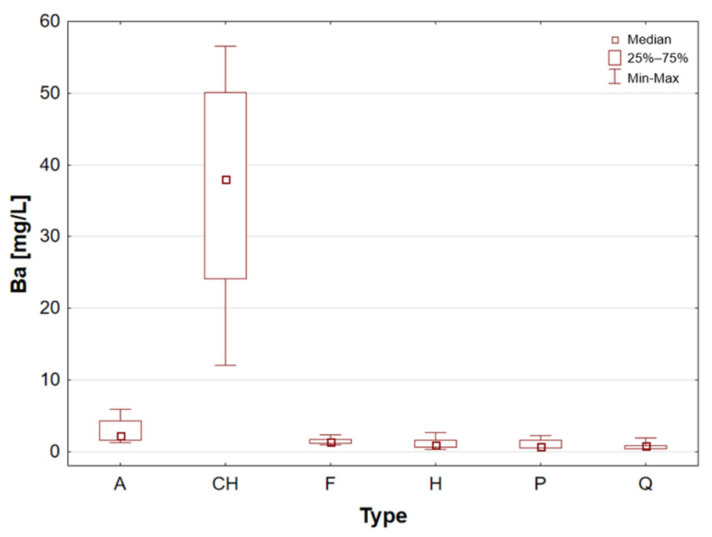
Box-and-whisker plot for Ba by type: A—amaranth grains, CH—chia seeds, F—flax seeds, H—hemp seeds, P—pumpkin seeds and Q—quinoa grains [mg/L].

A thorough examination of the results obtained for Ba reveals that chia seeds exhibit considerably higher concentrations in comparison to the other sample groups that were examined Table 1, Figure 1). The highest value was obtained for uncertified chia seeds (sample 10, 56.59 mg/L).

Elevated concentrations of heavy metals, particularly Cd and Pb, were detected in selected samples. In the flax seed group, all determined cadmium concentrations were above the limit of quantification. According to the applicable regulations, the general maximum permissible cadmium level for cereals and seeds is 0.10 mg/kg [35]. However, higher, product-specific limits are defined for certain commodities, including quinoa (0.15 mg/kg) and flax (linseed) (0.50 mg/kg) [35]. Based on these product-specific thresholds, no exceedance of the permissible cadmium levels was observed. The maximum cadmium concentration recorded for quinoa was 0.135 mg/L, while for flax seeds it reached 0.467 mg/L, remaining below the respective regulatory limits. In contrast, regulations regarding lead stipulate exclusively permissible content of this element for cereals and pulses. These values are consistent with those observed at 0.20 mg/L [35]. However, other types of seeds and grains are not mentioned. A thorough analysis of the results obtained for the samples that were analysed revealed that the highest Pb concentration was recorded in sample 37, which consists of pumpkin seeds, for which a certificate was not provided. Four additional elevated results, ranging from 1 to 1.70 mg/L, were obtained for amaranth samples, two samples of linseed, and a sample of pumpkin seeds that had been certified for organic food. Of the 43 samples analysed, only 13 were found to be free of Pb. Overall, the results are consistent with previously reported findings in the scientific literature.

For another potentially toxic metal, Ni, elevated median values were obtained in the chia seed, hemp seed, and pumpkin seed groups. The maximum value recorded was 2.982, which was obtained from sample 34, which was an uncertified pumpkin seed sample.

For other toxic elements, such as tin and titanium, the maximum values were recorded as 0.963 mg/L (sample 31, uncertified hemp seeds) and 2.110 mg/L (sample 6, uncertified chia seeds).

**Figure 2:**
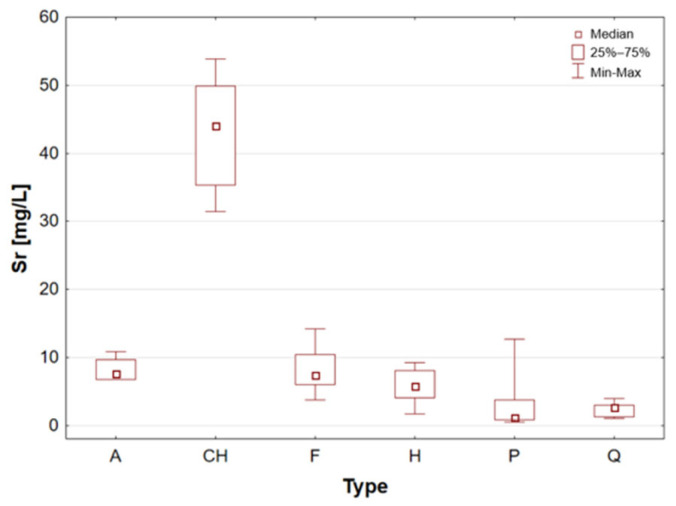
Box-and-whisker plot for Sr by type A—amaranth grains, CH—chia seeds, F—flax seeds, H—hemp seeds, P—pumpkin seeds and Q—quinoa grains [mg/L].

In a manner analogous to that observed in Ba, the median value of the chia seed sample group was found to be elevated in comparison to that of the other sample groups (Table 1, Figure 2). The maximum recorded value was 53.84 mg/L, which was obtained for sample 14, which had been certified.

The estimated dietary intake of cadmium was calculated for flax seed samples due to the elevated concentrations observed in this product group (sample 11). Based on the determined cadmium concentration and an assumed daily consumption of 30 g, the estimated weekly intake amounted to 1.40 µg/kg body weight/week. This value corresponds to approximately 56% of the tolerable weekly intake (TWI) for cadmium established by the European Food Safety Authority (EFSA) at 2.5 µg/kg body weight/week. Although the calculated intake does not exceed the EFSA safety threshold, it represents a substantial contribution to overall dietary cadmium exposure and may be of concern in the context of long-term consumption and cumulative exposure from multiple dietary sources [36].

For pumpkin seed samples, characterized by the highest determined lead concentration, the estimated weekly dietary exposure amounted to 8.05 µg/kg body weight/week, assuming a daily consumption of 30 g. Although no tolerable intake level has been established for lead, comparison with EFSA benchmark dose reference values indicates that the calculated exposure represents a substantial contribution to overall dietary lead intake [37]. These results suggest that regular consumption of pumpkin seeds may be relevant from a food safety perspective, particularly when cumulative exposure to lead from multiple dietary sources is considered.

#### 2.1.2. Macro- and Trace Elements

The samples of superfood seeds and grains were divided into groups according to their classification and subjected to statistical analysis. Table 2 presents the results of the analysis of the content of essential elements for individual groups of cereal and seed samples, divided into categories by type. The minimum, maximum and median values within each group are also indicated.

**Figure 3:**
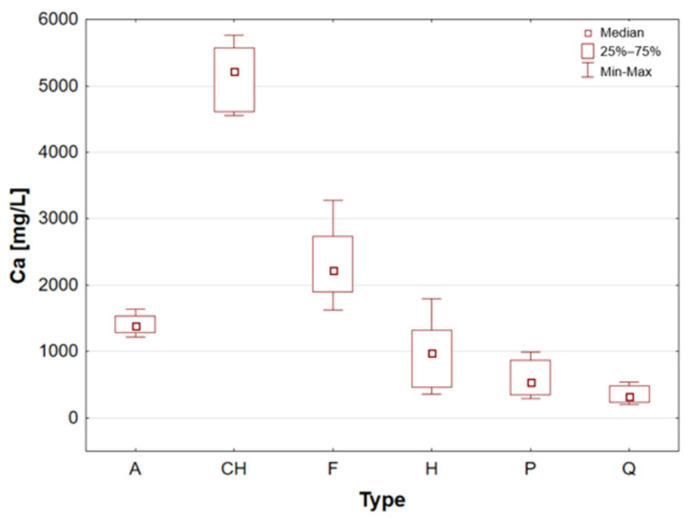
Box-and-whisker plot for Ca by type: A—amaranth grains, CH—chia seeds, F—flax seeds, H—hemp seeds, P—pumpkin seeds and Q—quinoa grains [mg/L].

**Figure 4:**
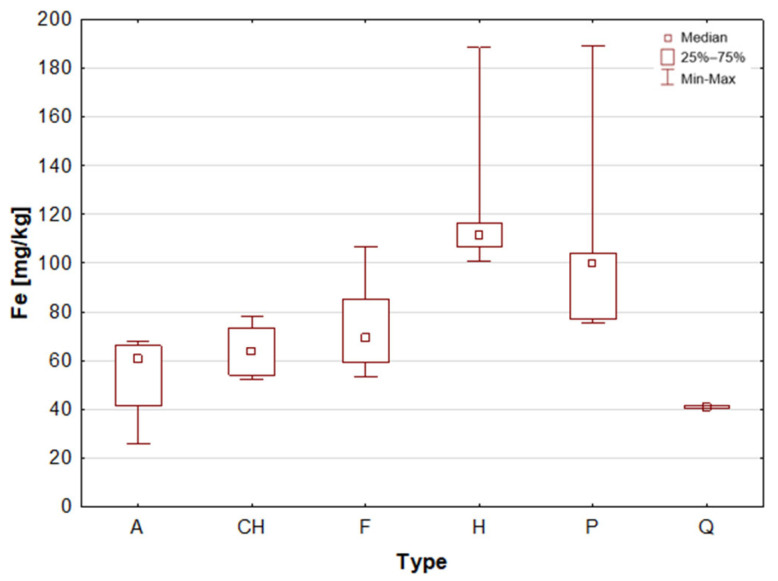
Box-and-whisker plot for Fe by type: A—amaranth grains, CH—chia seeds, F—flax seeds, H—hemp seeds, P—pumpkin seeds and Q—quinoa grains [mg/L].

Amaranth is a pseudocereal that possesses a high biological protein value (approximately 14 g per 100 g) and significant amounts of lysine, an amino acid that is typically limited in traditional cereal grains [10]. Amaranth has been shown to contain higher levels of protein and minerals in comparison to more commonly consumed cereals, thereby enhancing its overall nutritional value. Following thermal processing, the product exhibits adequate digestibility and bioavailability of nutrients. Amaranth is especially notable for its mineral content. The substance under scrutiny is a notable source of Ca, P and K [10,15,18,22]. Amaranth is a plant-based foodstuff that may be of particular benefit to those following a plant-based diet, due to its relatively high Fe content [15,18]. This is particularly relevant given that Fe deficiency risk is increased in such diets. Moreover, amaranth grains, known as the “golden grain” and “food of the past for the people of the future”, have been recognised by the National Academy of Sciences (NAS) as the best plant food for humans [22]. The obtained results are consistent with the extant literature data, as the analysed amaranth grain samples are characterised by a relatively high median value of Ca and Fe (Table 2, Figure 3 and Figure 4). The results obtained for amaranth are consistent with data reported in the available scientific literature. This agreement indicates that the elemental composition determined in the present study is comparable with previously published findings [26,38,39,40,41,42].

**Figure 5:**
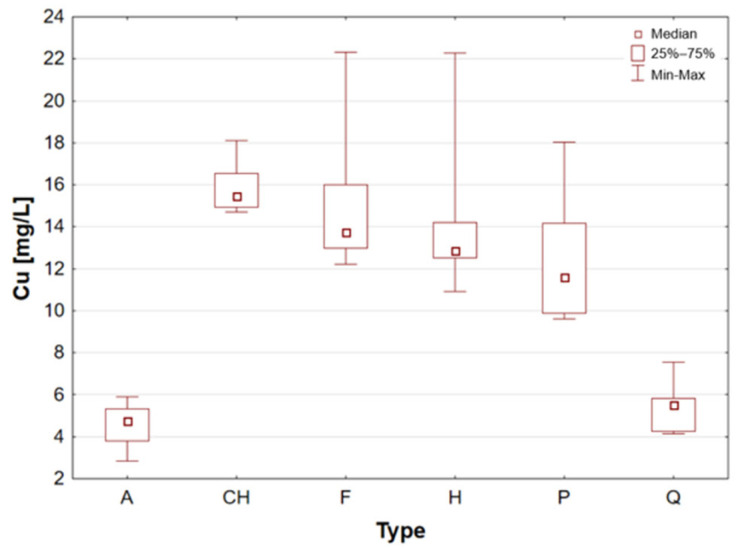
Box-and-whisker plot for Cu by type: A—amaranth grains, CH—chia seeds, F—flax seeds, H—hemp seeds, P—pumpkin seeds and Q—quinoa grains [mg/L].

Chia seeds are distinguished by their very high content of polyunsaturated fatty acids omega-3 (linolenic acid, 54–67%) and omega-6 (linoleic acid, 12–21%) and low in saturated fatty acids [1,16]. They constitute a significant natural source of soluble fibre, a nutritional component that has been demonstrated to enhance intestinal function and promote sustained satiety [1,4]. These elements are abundant in Ca, P, Mg, and Mn, which are vital for the strengthening of bones and the proper functioning of the nervous system [9,20,27]. In the presence of water, the chia seeds produce a transparent gelatinous substance known as chia mucilage. This substance has been shown to stabilise blood sugar levels and promote digestive processes within the human body. Chia seeds are distinguished by their high Ca and Mn content, with the potential to satisfy 10.7% of the daily magnesium requirement, 55% of the Mn requirement, 9.9% of the P requirement, and 7.9% of the Fe requirement [1]. Chia seeds were found to have a particularly high mineral content when compared to other samples. The analysis revealed that the chia seed sample exhibited the highest calcium concentration. The maximum recorded concentration was 5759 mg/L for sample 8 (Table 2). It is also noteworthy that the seeds exhibited a high degree of Cu content, with a maximum median Cu concentration value of 15.49 mg/L observed for this sample group (Table 1, Figure 5). The results obtained for chia seeds are also consistent with data reported in the available scientific literature. Among the analysed samples, chia seeds represent one of the most commonly consumed pseudocereals, alongside flax seeds, which further supports the relevance of the obtained findings [26,27,38,43,44,45].

**Figure 6:**
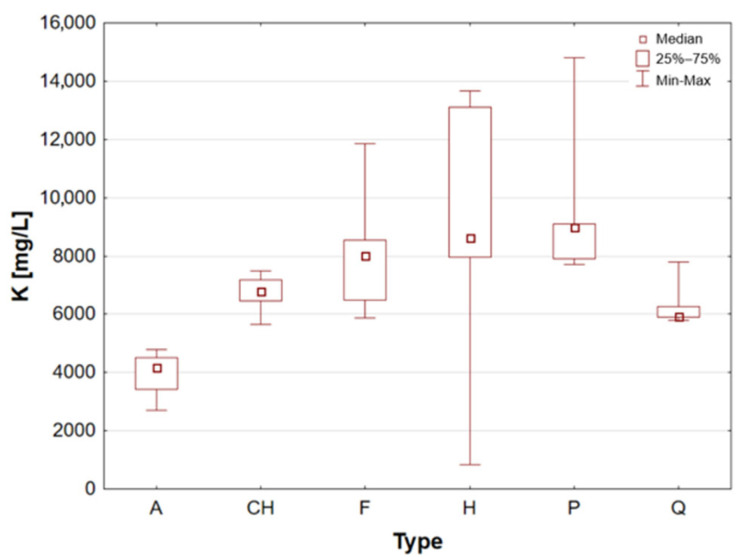
Box-and-whisker plot for K by type: A—amaranth grains, CH—chia seeds, F—flax seeds, H—hemp seeds, P—pumpkin seeds and Q—quinoa grains [mg/L].

**Figure 7:**
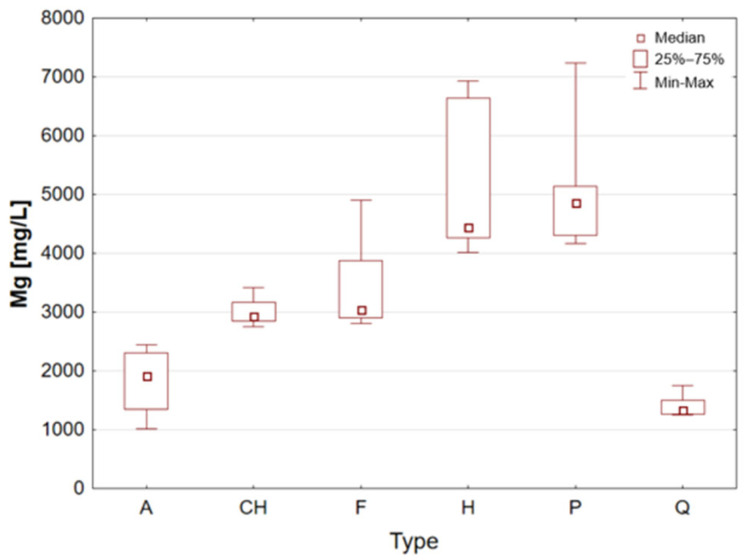
Box-and-whisker plot for Mg by type: A—amaranth grains, CH—chia seeds, F—flax seeds, H—hemp seeds, P—pumpkin seeds and Q—quinoa grains [mg/L].

**Figure 8:**
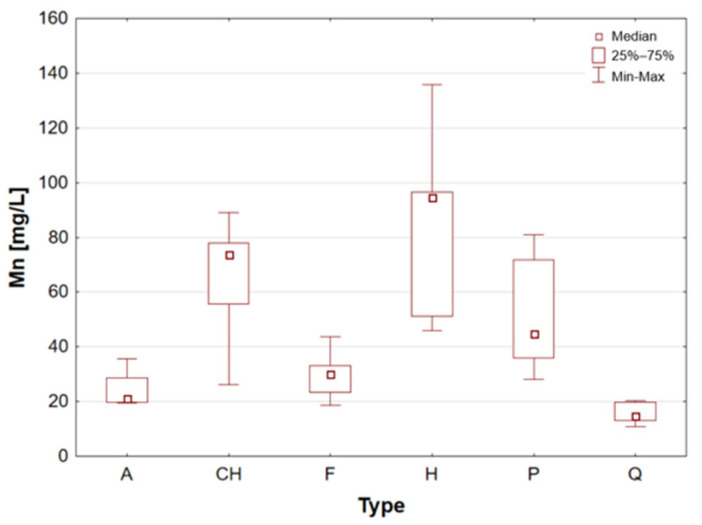
Box-and-whisker plot for Mn by type: A—amaranth grains, CH—chia seeds, F—flax seeds, H—hemp seeds, P—pumpkin seeds and Q—quinoa grains [mg/L].

Flax seed is considered to be one of the most significant plant sources of omega-3 fatty acids (ALA) and lignans, which have been demonstrated to possess antioxidant properties and to support hormonal balance [1,4,10]. The product contains a high amount of fibre, including soluble fibre, which supports gut microbiota and alleviates digestive issues [9]. The mineral composition of flax seed is notable, with high concentrations of magnesium, phosphorus, copper, and B vitamins, which have been demonstrated to support metabolic processes and nervous system function [1,30]. It has been demonstrated that whole ground flax seed is the most digestible form, as it frequently passes through the digestive system without undergoing any alteration. Flax seed has also been demonstrated to assist in the regulation of cholesterol levels, a property that can be attributed to the presence of plant mucilage’s [1]. Flax seeds, akin to chia seeds, are distinguished by their elevated calcium content, exhibiting a median concentration of 2220 mg/L within this particular sample group. Furthermore, these samples have been found to contain substantial quantities of Fe, Cu, K, Mg and Mn (see Figure 4, Figure 5, Figure 6, Figure 7 and Figure 8). These findings are consistent with data reported in the available scientific literature for flax seeds [26,28,30,38,43,44,46].

**Figure 9:**
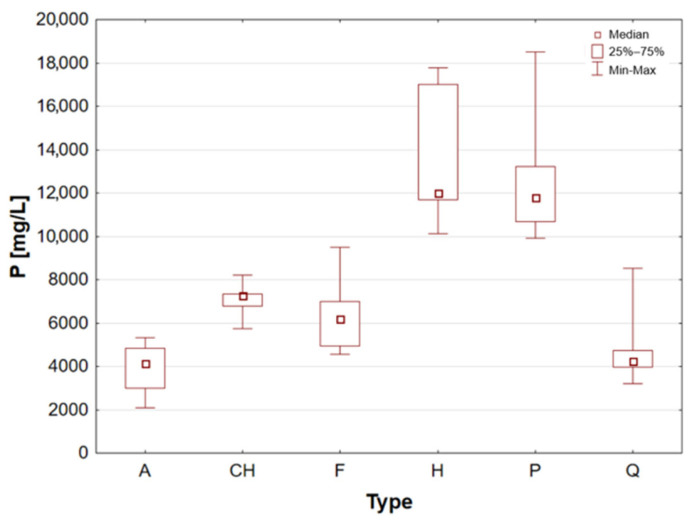
Box-and-whisker plot for P by type: A—amaranth grains, CH—chia seeds, F—flax seeds, H—hemp seeds, P—pumpkin seeds and Q—quinoa grains [mg/L].

**Figure 10:**
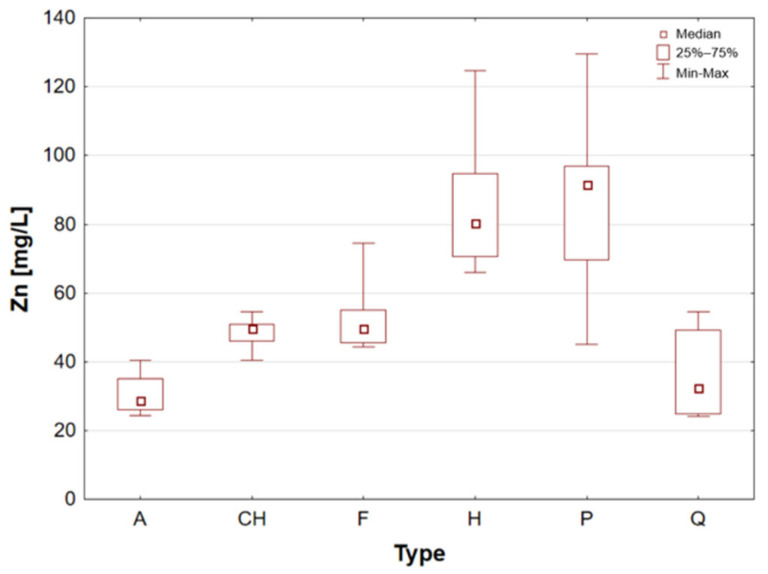
Box-and-whisker plot for Zn by type: A—amaranth grains, CH—chia seeds, F—flax seeds, H—hemp seeds, P—pumpkin seeds and Q—quinoa grains [mg/L].

Hemp seeds provide high-quality protein with a complete set of amino acids, distinguishing them from other seeds [13,17,21]. A significant proportion (exceeding 90%) of unsaturated fatty acids can be found in hempseed, which is crucial for the stability of omega-6 and omega-3 fatty acids, thereby ensuring a balanced intake of essential nutrients [12,13,25]. They are rich in Fe, Mg, Zn, Mn, and vitamin E, a powerful antioxidant [13,29]. They are highly digestible and have a mild, nutty flavour, making them a great addition to the diets of athletes and those following plant-based diets [14,17,19]. The analysis demonstrated that the elemental composition of hemp seeds is comparable to that of pumpkin seeds, as evidenced by the median concentrations of almost all the macro- and trace elements studied, which exhibited a high degree of similarity (Table 2, Figure 3, Figure 4, Figure 5, Figure 6, Figure 7, Figure 8, Figure 9 and Figure 10). It is noteworthy that hemp seed samples exhibited the highest median concentrations of Fe (111.3 mg/L), Mn (94.57 mg/L), P (12,010 mg/L) and S (3302 mg/L) among the samples analysed. This elemental composition, rich in nutrients, has led to the seeds gaining significant popularity among consumers, particularly those who adhere to vegan diets. The results obtained for hemp seeds are consistent with data reported in the available scientific literature [29,43].

According to the extant literature, pumpkin seeds provide plant protein and healthy unsaturated fats that support heart function [2]. Furthermore, they constitute a significant source of zinc, a mineral that has been demonstrated to support immunity, skin health, and hormonal balance. Furthermore, they are abundant in Mg, P, Cu, Mn, and Fe, which support muscle, bone, and nervous system function [11]. Furthermore, these products have been found to contain phytosterols, which have been demonstrated to have a beneficial effect on cholesterol levels. The presence of vitamin E and carotenoids in the sample is indicative of antioxidant and anti-inflammatory properties [2,9]. A thoroughgoing analysis of the obtained results reveals that the pumpkin seed group is distinguished by an increased concentration of macronutrients such as K, Mg, P, and S (Table 2, Figure 6, Figure 7 and Figure 9), as well as micronutrients such as Fe, Cu, and Zn (Table 2, Figure 4, Figure 5 and Figure 10). Among the samples analysed, pumpkin seeds were found to have the highest zinc concentration. The median Zn concentration was found to be 91.46 mg/L, with a maximum value of 129.6 mg/L being obtained. For pumpkin seeds, the obtained results correspond well with data available in the scientific literature. The observed agreement indicates that the elemental composition determined in this study is comparable to values previously reported for this widely consumed plant source [46].

Quinoa is renowned for its high supply of high-quality protein, thus classifying it as one of the most valuable plant-based alternatives to animal products. Its low glycaemic index contributes to the stabilisation of blood glucose levels. The subject is a notable source of Mg, Fe, P, Zn, and antioxidants, including flavonoids [1,22]. Furthermore, the presence of saponins, which possess anti-inflammatory properties, is notable, though these are generally rinsed out prior to consumption. Quinoa is naturally devoid of gluten, thus rendering it a valuable constituent of elimination diets. Statistical analyses indicated that quinoa grains exhibited the least optimal elemental composition. However, this does not imply that they are devoid of value, as they encompassed a number of essential elements, including Ca, Fe, K, Mg, P, and S (Table 2, Figure 3, Figure 4, Figure 6, Figure 7 and Figure 9). A thorough examination of the obtained results indicates that the elemental compositions of the pseudocereals under study, i.e., amaranth and quinoa, are comparable. However, amaranth grains were found to contain higher concentrations of Ca, Fe, and Mg, while quinoa grains contained significantly higher levels of K. The results obtained for quinoa are consistent with values reported in the available scientific literature. This agreement suggests that the elemental composition determined in the present study is comparable to previously published data for quinoa, a pseudocereal of growing nutritional significance [26,38,39,41,42,43,47].

### 2.2. Intergroup Comparisons and Statistical Significance

Table 3 presents the elements and groups of tested samples between which statistically significant differences were found. A number of differences were identified for a number of the elements analysed. A detailed analysis of the content of individual elements is presented in Section 2.1, while this subsection will focus on the important differences between the tested sample groups.

Statistically significant differences (*p* < 0.05) were identified for most elements, indicating measurable variability among the analysed matrices. Macro- and microelements such as Ca, K, Mg, Fe, Mn, P, S, and Zn showed particularly pronounced differences between selected groups, while for trace elements including Cd, Co, Cr, Mo, and Ni, significant differences were limited to specific comparisons. In several cases, concentrations below the limit of quantification were observed in certain groups, further emphasizing differences in elemental distribution. These results suggest that the elemental composition is influenced by the sample type, resulting in distinct concentration patterns across the analysed groups. A graphical illustration of the statistically significant differences is additionally provided in the box-and-whisker plots presented in the preceding sections for selected elements (Figure 1, Figure 2, Figure 3, Figure 4, Figure 5, Figure 6, Figure 7, Figure 8, Figure 9 and Figure 10), where the distribution of the obtained results and the variability between sample groups can be visually assessed.

### 2.3. Principal Component Analysis (PCA)

PCA analysis was performed to assess whether samples belonging to the same groups exhibited clear clustering. This analysis allowed for the verification of the internal consistency of each of the sample categories studied.

**Figure 11:**
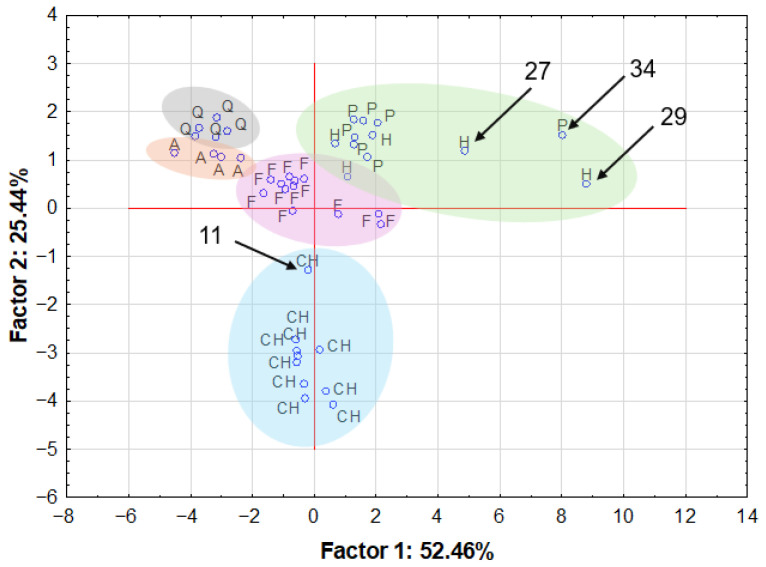
Projection of the cases on the factor-plane for the 43 samples of studied superfood samples divided by type: A—amaranth, CH—chia seeds, F—flax seed, H—hemp seeds, P—pumpkin seeds and Q—quinoa, after Ag, Al, Cd, Co, Pb, Ti and Sn reduction (ellipses representing sample groups were added manually).

As illustrated in Figure 11, the cases have been projected onto the factor plane for the analysed samples following the reduction of elements whose impact on the principal component analysis was negligible. The initial model explained only 53.6% of the total variance. Following the application of nonparametric statistical tests and a preliminary PCA assessment, a decision was made to exclude selected elements (Ag, Al, Cd, Co, Pb, Ti, and Sn). This was justified by the lack of statistically significant differences between the analysed groups for some of these elements, as well as by low contributions to the first two principal components (loadings below 0.5) for others. This approach facilitated the identification of multiple sample groups, namely chia seed samples (CH—blue), amaranth grain samples (A—orange) and quinoa grain samples (A and Q—grey), hemp and pumpkin seed samples (H and P—green), and flax seed samples (F—purple). The total explained variance in this case after reduction was almost 78%. Principal component analysis demonstrated clear clustering of amaranth and quinoa grain samples in the score space, with their close proximity reflecting similar contributions of elemental variables. As outlined earlier, these seeds are classified as pseudocereals, and their elemental composition exhibits notable similarities, though they are comparatively deficient in macro elements when compared to the other grains and seeds analysed. In a similar manner, the samples from the hemp seed and pumpkin seed groups are combined to form a single group. However, these samples are distinguished by a lower Ca content and higher Fe, K, Mg, P, and Zn content in comparison to the samples from the chia and flax seed groups. The exceptions to this are samples 27 and 29 from the hemp group and sample 34 from the pumpkin seed group, which are clearly outliers. Consequently, the elemental composition of these samples deviates from that of the coherent group. The three samples in question are distinguished by their significantly elevated concentrations of Ca, Cu, Fe, K, Mg, Mn, P, S and Zn when compared to the other samples from either group. The situation is analogous with the chia seed group, in that the majority of the samples constitute one group, with one exception—sample 11. This sample, unlike the others in this group, is characterised by lower Ba and Ca content, but higher Fe, K, Mn, Mo and P content. The flax seed sample group is characterised by its compact structure, situated between the hemp-pumpkin seed group (H,P) and the chia seed group (CH).

### 2.4. Verification of the Composition of the Studied Samples According to the Information Provided on the Product Labels

The composition of some superfood products is specified on the product’s packaging. This includes information on the content of elements that are essential for the healthy development of living organisms. A comparison was made between the values declared by the manufacturers and the values obtained during the analysis. The values under discussion are summarised and presented in Table 4.

The majority of the results obtained for the analysed products fall within the permissible deviation from the declared value on the packaging, as established by relevant EU regulations [48,49]. With regard to minerals, these values range from—35% to +45% of the value indicated on the packaging [48]. It was determined that only samples 5, 11, 21, and 40 exhibited significant discrepancies with the values declared by food manufacturers. Specifically, sample 5 (chia seeds) exhibited an inadequate level of K (61.5% of the declared value) and an excessive level of Mn (155% of the declared value). It is also worthy of note that this is a certified sample. In contrast, Sample 11 (chia seeds, uncertified sample) exhibited an excessively elevated K content (184% of the declared value). The most significant deviations from the declared values were observed in sample 21 (flax seeds), which exhibited elevated concentrations of Cu and Mn (346% and 250% of the declared value, respectively) and reduced levels of Fe and Zn (53.2% and 59.7% of the declared value, respectively). Conversely, sample 40 (quinoa grains) exhibited an inadequate Mg content. This value was found to be only 63.9% of the declared value on the food product packaging.

### 2.5. Summary

The term “superfoods” refers to a rapidly expanding category of food products that have garnered heightened consumer interest on a global scale in recent years. The growing nutritional awareness of consumers, coupled with the pursuit of products that offer high nutritional value, has led to their integration into daily diets. Superfoods are distinguished by their rich composition of vitamins, minerals, dietary fibre, unsaturated fatty acids, and numerous bioactive compounds. A significant proportion of these products can be considered a valuable addition to elimination diets, including gluten-free, vegetarian, or vegan diets, due to their high content of plant protein and other key nutrients. A further salient aspect pertains to the fact that a number of products classified as superfoods are plants that have been known and utilised for millennia in diverse cultures, and which are now witnessing a resurgence in contemporary nutrition. The rediscovery of these foods can be attributed to two key factors. Firstly, scientific research has confirmed their beneficial properties. Secondly, trends in the food industry have promoted the concept of naturalness and minimal processing. A further characteristic of superfoods is their considerable culinary versatility, which facilitates their wide use in various food groups. Consequently, they constitute a significant element of contemporary nutritional trends, combining tradition with current knowledge in dietetics and food science.

The analyses performed confirm the rich elemental composition of the studied grain and seed groups. In consideration of the results obtained, the following ranges of selected elemental contents in the analysed samples can be presented (taking into account the median concentration values):

Ca:

chia >> flax > amaranth > hemp > pumpkin > quinoa

Mg:

pumpkin > hemp > chia~flax > amaranth > quinoa

K:

pumpkin > hemp > flax > chia > quinoa > amaranth

P:

hemp~pumpkin > chia > flax > amaranth~quinoa

Fe:

hemp > pumpkin > flax~chia~amaranth > quinoa

Mn:

hemp > chia > pumpkin > flax > amaranth > quinoa

Zn:

pumpkin > hemp > chia = flax > quinoa > amaranth

## 3. Materials and Methods

### 3.1. Samples

In the present study, the elemental composition of 43 samples of seeds and grains was analysed. As demonstrated in Table 4, the samples that were examined and the respective abbreviations are presented in the following list. The samples were procured from brick-and-mortar stores, as well as online retailers, including health food stores, in Poland. The samples that were analysed consisted of ready-to-eat seeds and grains that had been packaged in lightweight packaging from various manufacturers. The product packaging contained information pertaining to the elemental composition, provenance, and any applicable certifications. However, it was observed that a number of the products analysed did not have additional labelling. It should be noted that the sampling design was based on market-available products, with a single sample analysed per product. Therefore, the results may not fully reflect batch-to-batch variability and should be interpreted as indicative rather than representative of all products on the market.

Of the samples that were analysed, 12 were identified by the presence of valid certificates, which are listed in Table 5. These certificates are for foodstuffs originating from outside the European Union, and the numbers correspond to the relevant certification bodies in Poland. It is evident that each certified product has been distinguished by the relevant green leaf symbol.

### 3.2. Sample Preparation

Samples were collected from a single package, crushed, and then weighed on an analytical balance (OHAUS, Corporation, Parsippany, NJ, USA) to the nearest 0.00001 g into disposable containers (approximately 0.15 g of sample was weighed). For the purpose of the digestion process, a quantity of 2 mL of HNO_3_ (Suprapur, Merck, Darmstadt, Germany) with a percentage range of 69.0–70.0% was added to each test tube. This was followed by the addition of 0.5 mL of 30% H_2_O_2_ (Chempur, Piekary Śląskie, Poland) also using automatic pipettes. The test tubes were then sealed with Teflon stoppers, and the prepared test tubes were subjected to wet digestion using a microwave digester (UltraWave, Milestone, Milan, Italy). In order to minimise the risk of error, each sample was mineralised in three independent replicates. Blank samples were prepared in an analogous manner. Table 6 presents the conditions of the sample digestion process. Following mineralisation, the resulting solutions were quantitatively transferred to volumetric flasks, followed by the addition of an internal standard (Yb, PlasmaCAL, Villebon-sur-Yvette, France). Subsequently, the samples were diluted to a final volume of 25 mL. The reference materials used in this study were Certified Rice Flour (1568b, National Institute of Standards and Technology, Gaithersburg, MD, USA) and INCT-MPH-2, Mixed Polish Herbs (Institute of Nuclear Chemistry and Technology, Warsaw, Poland).

### 3.3. ICP-OES (Inductively Coupled Plasma–Optical Emission Spectrometry)

The determination of elemental composition was performed using a dual-view ICP-OES spectrometer iCAP™ 7400 from Thermo Scientific™ (Waltham, MA, USA). The essential operating settings of the instrument applied during the procedure are listed in Table 7. All working standard solutions were obtained by dilution of the respective stock solutions with ultrapure water (Milli-Q, Millipore, Bedford, MA, USA), which was used throughout the entire analytical procedure. Prior to the commencement of the analytical procedure, calibration standards for all target elements were prepared. A multi-element ICP standard solution (100 mg/L) supplied by CPAchem (Stara Zagora, Bulgaria) was utilised, in conjunction with single-element standards S (ICP grade, Analytika, Prague, Czech Republic) and P (ICP grade, PlasmaCAL, Courtaboeuf, France), each at a concentration of 1000 mg/L. Calibration curves were constructed over a concentration range extending from the limit of quantification (LOQ) to the highest calibration standard.

Table 8 lists the analytical wavelengths selected for ICP-OES measurements, together with the analysed elements and their corresponding limits of quantification. Linearity of the method was evaluated using multi-element calibration standards in the concentration range of 0.01–10.0 mg/L, showing satisfactory determination coefficients (R^2^ ≥ 0.998) for all analytes (Table 8). To express the method sensitivity in relation to the solid samples, LOQ values obtained in solution (mg/L) were converted to mg/kg, taking into account the final volume of the digest and the sample mass. Calculations were performed assuming a representative sample mass of 0.15 g and a final volume of 25 mL. Minor variations in sample mass were considered negligible. Each sample was analysed in triplicate to ensure analytical repeatability. To verify the accuracy of the procedure, certified reference materials that had undergone prior mineralisation were prepared and analysed in duplicate. These measurements confirmed the reliability of both the sample preparation protocol and the instrumental method. Ytterbium was used as an internal standard throughout the analysis, enabling continuous correction of potential signal drift as well as matrix-induced fluctuations, and ensuring stable analytical performance during ICP-OES determinations.

Table 9 presents the analytical results obtained for the certified reference materials analyzed in this study. The measured concentrations were generally consistent with the certified values for all investigated elements. The calculated recoveries ranged from 87.5% to 110.3%, indicating acceptable analytical performance of the proposed method. The method also demonstrated good precision, with RSD values below 6%.

### 3.4. Data Analysis

Statistical and multivariate analyses were performed using Statistica software, version 12.5 (New York, NY, USA).

In order to ascertain whether the data distribution for all analysed samples conformed to a normal distribution at the assumed significance level (*p* = 0.05), the Shapiro–Wilk and Kolmogorov–Smirnov tests were applied. The findings suggested that the assumption of normality had not been met. Consequently, subsequent analyses were executed employing statistical methodologies appropriate for non-normally distributed data, namely the non-parametric Kruskal–Wallis test. The objective of the test was to evaluate the statistical significance of variations in the established elemental levels between distinct groups, with regard to the parameter of product type.

Principal Component Analysis (PCA) was applied as an exploratory multivariate technique to reduce data dimensionality while retaining the maximum possible variance. The method transforms the original correlated variables into a smaller set of orthogonal principal components, facilitating the identification of patterns and relationships within the dataset. The purpose of the present study was to utilise the PCA in order to evaluate the following: firstly, the similarities between the samples; secondly, the separation of the samples into groups; and thirdly, the underlying structure of the samples.

## 4. Conclusions

The analyses confirmed that the investigated superfood grains and seeds are characterized by a rich and diverse elemental composition, with clear differences observed between product groups. Principal component analysis revealed distinct clustering patterns, indicating similarities between amaranth and quinoa grains as well as between hemp and pumpkin seeds, while chia and flax seeds formed separate groups. Verification of manufacturers’ declarations showed that, in most cases, the analytically determined elemental contents were consistent with the values declared on product packaging within the permissible deviation ranges. However, notable discrepancies were identified for selected samples, indicating that declared values do not always fully reflect the actual elemental composition. The assessment of toxic elements demonstrated that, although elevated concentrations of cadmium and lead were observed in specific product groups, no exceedance of applicable regulatory limits was identified when appropriate reference values were applied. Overall, the results highlight the nutritional potential of superfoods while emphasizing the importance of independent analytical verification of their elemental composition to ensure food safety and consumer confidence.

## Figures and Tables

**Table 1:** Content of the toxic elements in the analysed samples, divided by type of seeds and grains, along with the minimum, maximum and median values within the group: A—amaranth grains, CH—chia seeds, F—flax seeds, H—hemp seeds, P—pumpkin seeds and Q—quinoa grains [mg/L].

Element	Amaranth Grains A (n = 4)	Chia Seeds CH (n = 10)	Flax Seeds F (n = 12)	Hemp Seeds H (n = 5)	Pumpkin Seeds P (n = 7)	Quinoa Grains Q (n = 5)
	**Min–Max** **(Median)**	**Min–Max** **(Median)**	**Min–Max** **(Median)**	**Min–Max** **(Median)**	**Min–Max** **(Median)**	**Min–Max** **(Median)**
Ag	<LOQ–0.145 (<LOQ)	<LOQ–0.300 (<LOQ)	<LOQ *	<LOQ *	<LOQ–0.317 (<LOQ)	<LOQ *
Al	7.449–25.37 (15.71)	9.202–39.36 (23.83)	21.04–52.37 (29.79)	<LOQ–32.89 (22.96)	18.93–37.18 (29.28)	10.13–16.17 (12.67)
Ba	1.260–5.874 (2.224)	12.01–56.59 (38.02)	0.913–2.368 (1.335)	0.253–2.649 (0.927)	0.441–2.244 (0.750)	0.356–1.897 (0.760)
Cd	<LOQ–0.137 (<LOQ)	<LOQ *	0.134–0.467 (0.137)	<LOQ *	<LOQ–0.138 (<LOQ)	<LOQ–0.135 (0.134)
Ni	0.055–0.219 (0.217)	0.382–2.682 (1.621)	1.026–2.499 (0.539)	0.371–2.836 (1.341)	0.536–2.982 (1.225)	0.217–0.536 (0.325)
Pb	<LOQ–1.702 (0.215)	<LOQ–0.884 (0.295)	<LOQ–1.374 (0.381)	<LOQ–0.055 (0.053)	<LOQ–2.681 (0.056)	<LOQ–0.380 (<LOQ)
Sn	<LOQ–0.438 (0.356)	<LOQ–0.765 (0.275)	<LOQ–0.756 (0.273)	<LOQ–0.963 (0.111)	<LOQ–0.772 (0.445)	0.109–0.750 (0.333)
Sr	6.737–10.85 (7.572)	31.47–53.84 (44.08)	3.704–14.18 (7.363)	1.636–9.217 (5.794)	0.496–12.70 (1.149)	1.004–3.902 (2.604)
Ti	0.110–0.438 (0.271)	0.421–2.110 (0.601)	0.601–1.413 (0.994)	0.107–0.424 (0.123)	0.110–1.898 (0.274)	<LOQ–0.109 (0.089)

<LOQ—below limit of quantification, *—all results obtained for all samples in the given group were <LOQ

**Table 2:** Content of the essential elements (macro- and trace elements) in the analysed samples, divided by type of seeds and grains, along with the minimum, maximum and median values within the group: A—amaranth grains, CH—chia seeds, F—flax seeds, H—hemp seeds, P—pumpkin seeds and Q—quinoa grains [mg/L].

Element	Amaranth Grains A (n = 4)	Chia Seeds CH (n = 10)	Flax Seeds F (n = 12)	Hemp Seeds H (n = 5)	Pumpkin Seeds P (n = 7)	Quinoa Grains Q (n = 5)
	**Min–Max** **(Median)**	**Min–Max** **(Median)**	**Min–Max** **(Median)**	**Min–Max** **(Median)**	**Min–Max** **(Median)**	**Min–Max** **(Median)**
Ca	1223–1639 (1388)	4553–5759 (5219)	1627–3274 (2220)	358.4–1792 (981.5)	297.3–990.8 (541.4)	199.3–537.3 (326.6)
Co	0.134–0.137 (0.137)	<LOQ–0.138 (0.066)	0.134–0.962 (0.214)	<LOQ–0.139 (<LOQ)	<LOQ–0.138 (<LOQ)	<LOQ–0.145 (<LOQ)
Cr	0.161–0.165 (0.164)	<LOQ–0.167 (0.163)	0.159–0.165 (0.163)	0.159–1.666 (0.322)	0.164–0.651 (0.331)	<LOQ–0.163 (<LOQ)
Cu	2.863–5.888 (4.745)	14.71–18.13 (15.49)	12.23–22.31 (13.76)	10.92–22.29 (12.89)	9.615–18.03 (11.59)	4.153–7.563 (5.507)
Fe	25.91–67.76 (60.77)	52.46–78.39 (63.75)	53.65–106.5 (69.24)	100.8–188.6 (111.3)	75.26–189.0 (99.70)	40.26–41.51 (40.90)
K	2697–4793 (4187)	5654–7484 (6786)	5863–11,840 (8013)	846.2–13,660 (8626)	7724–14,810 (8996)	5797–7797 (5921)
Mg	1015–2444 (1923)	2758–3417 (2926)	2807–4910 (3047)	4018–6934 (4450)	4167–7232 (4864)	1250–1753 (1333)
Mn	19.60–35.66 (21.05)	26.25–89.17 (73.79)	18.78–43.59 (29.97)	45.90–135.9 (94.57)	28.22–80.88 (44.69)	11.00–20.32 (14.65)
Mo	0.214–0.549 (0.384)	0.217–0.707 (0.385)	0.371–1.374 (0.539)	0.848–1.388 (1.180)	0.221–2.625 (1.058)	0.185–0.379 (0.214)
P	2085–5308 (4136)	5746–8208 (7260)	4559–9493 (6212)	10,110–17,790 (12,010)	9913–18,490 (11,800)	3200–8531 (4241)
S	938.0–1751 (1689)	2541–3157 (2743)	1835–3397 (2117)	2436–4983 (3302)	2442–5093 (2855)	1142–1371 (1215)
Zn	24.44–40.37 (28.72)	40.54–54.63 (49.69)	44.34–74.53 (49.69)	65.90–124.7 (80.26)	45.10–129.6 (91.46)	24.20–54.57 (32.51)

<LOQ—below limit of quantification.

**Table 3:** Elements with statistically significant differences and the groups between which they were observed along with the minimum, maximum and median values within the group: A—amaranth grains, CH—chia seeds, F—flax seeds, H—hemp seeds, P—pumpkin seeds and Q—quinoa grains [mg/L].

Element	Type	N	Mean	Median	Min.	Max.	SD	Statistically Significant Differences
Al	A	4	16.06	15.71	7.449	25.37	7.604	F/Q
	CH	10	24.70	23.83	9.202	39.36	10.66	
	F	12	30.62	29.79	21.04	52.37	8.924	
	H	5	17.99	22.96	<LOQ	32.89	15.30	
	P	7	28.92	29.28	18.93	37.18	5.510	
	Q	5	13.04	12.67	10.13	16.17	2.416	
Ba	A	4	2.896	2.224	1.260	5.874	2.057	CH/H CH/F CH/P CH/Q
	CH	10	36.97	38.02	12.01	56.59	15.12	
	F	12	1.423	1.335	0.913	2.368	0.411	
	H	5	1.206	0.927	0.253	2.649	0.950	
	P	7	1.000	0.750	0.441	2.244	0.673	
	Q	5	0.857	0.760	0.356	1.897	0.618	
Ca	A	4	1409	1388	1223	1639	174.4	CH/F, CH/P, CH/Q, F/P, F/Q
	CH	10	5172	5219	4553	5759	471.6	
	F	12	2322	2220	1627	3274	563.1	
	H	5	981.6	981.5	358.4	1792	599.1	
	P	7	585.8	541.4	297.3	990.8	260.2	
	Q	5	355.6	326.6	199.3	537.3	148.6	
Cd	A	4	0.035	<LOQ	<LOQ	0.137	0.068	CH/F, H/F, F/P
	CH	10	<LOQ					
	F	12	0.244	0.137	0.134	0.467	0.144	
	H	5	<LOQ					
	P	7	0.021	<LOQ	<LOQ	0.138	0.052	
	Q	5	0.101	0.134	<LOQ	0.135	0.058	
Co	A	4	0.136	0.137	0.134	0.137	0.002	F/P
	CH	10	0.068	0.066	<LOQ	0.138	0.071	
	F	12	0.299	0.214	0.134	0.962	0.254	
	H	5	0.056	<LOQ	<LOQ	0.139	0.075	
	P	7	0.021	<LOQ	<LOQ	0.138	0.052	
	Q	5	0.056	<LOQ	<LOQ	0.145	0.076	
Cr	A	4	0.163	0.164	0.161	0.165	0.002	H/Q, F/P, P/Q
	CH	10	0.147	0.163	<LOQ	0.167	0.051	
	F	12	0.163	0.163	0.159	0.165	0.002	
	H	5	0.585	0.322	0.159	1.666	0.615	
	P	7	0.328	0.331	0.164	0.651	0.162	
	Q	5	0.044	<LOQ	<LOQ	0.163	0.070	
Cu	A	4	4.560	4.745	2.863	5.888	1.253	CH/A, CH/Q, F/A, F/Q
	CH	10	15.78	15.49	14.71	18.13	1.086	
	F	12	14.86	13.76	12.30	22.31	2.844	
	H	5	14.57	12.89	10.92	22.29	4.474	
	P	7	12.38	11.59	9.615	18.03	2.918	
	Q	5	5.461	5.507	4.153	7.563	1.389	
Fe	A	4	53.80	60.77	25.91	67.76	19.18	CH/H, H/A, H/Q, P/Q
	CH	10	64.31	63.75	52.46	78.39	9.783	
	F	12	73.78	69.24	53.65	106.5	17.84	
	H	5	124.7	111.3	100.8	188.6	36.17	
	P	7	106.3	99.70	75.26	189.0	38.33	
	Q	5	40.92	40.90	40.26	41.51	0.593	
K	A	4	3966	4187	2697	4793	893.6	H/A, F/A, H/Q, P/Q
	CH	10	6732	6786	5654	7484	516.9	
	F	12	7967	8013	5863	11,840	1807	
	H	5	8846	8626	846.2	13,660	5154	
	P	7	9395	8996	7724	14,810	2462	
	Q	5	6339	5921	5797	7797	834.0	
Mg	A	4	1826	1923	1015	2444	628.8	H/A, H/Q, P/A, P/Q
	CH	10	3004	2926	2758	3417	221.1	
	F	12	3422	3047	2807	4910	830.2	
	H	5	5262	4450	4018	6935	1406	
	P	7	5070	4864	4167	7232	1026	
	Q	5	1420	1333	1250	1753	212.4	
Mn	A	4	24.34	21.05	19.60	35.66	7.606	CH/F, CH/A, CH/Q, H/F, H/A, H/Q, P/Q
	CH	10	67.63	73.79	26.25	89.17	18.30	
	F	12	30.02	29.97	18.78	43.59	7.684	
	H	5	84.80	94.57	45.90	135.9	37.04	
	P	7	50.33	44.69	28.22	80.88	19.37	
	Q	5	15.77	14.65	11.00	20.323	4.089	
Mo	A	4	0.383	0.384	0.214	0.549	0.137	H/Q, P/Q
	CH	10	0.445	0.385	0.217	0.707	0.171	
	F	12	0.611	0.539	0.371	1.374	0.304	
	H	5	1.123	1.180	0.848	1.388	0.207	
	P	7	1.245	1.058	0.221	2.625	0.862	
	Q	5	0.239	0.214	0.185	0.379	0.079	
Ni	A	4	0.177	0.217	0.055	0.219	0.081	CH/A, P/A, F/Q
	CH	10	1.574	1.621	0.382	2.682	0.639	
	F	12	1.570	1.373	1.026	2.499	0.496	
	H	5	1.478	1.341	0.371	2.836	0.987	
	P	7	1.631	1.225	0.536	2.982	0.937	
	Q	5	0.359	0.325	0.217	0.536	0.153	
P	A	4	3916	4136	2085	5308	1353	H/F, H/A, H/Q, F/P, A/P, Q/P
	CH	10	7134	7260	5746	8208	664.9	
	F	12	6264	6212	4559	9493	1438	
	H	5	13,720	12,010	10,110	17,790	3442	
	P	7	12,530	11,800	9913	18,490	2825	
	Q	5	4935	4241	3200	8531	2086	
S	A	4	1517	1689	938.0	1751	388.2	CH/Q, H/A, H/Q, A/P, P/Q
	CH	10	2784	2743	2541	3157	170.4	
	F	12	2369	2117	1835	3397	587.1	
	H	5	3360	3302	2436	4983	981.8	
	P	7	3147	2855	2442	5093	906.8	
	Q	5	1240	1214	1142	1371	84.10	
Sr	A	4	8.183	7.572	6.737	10.85	1.943	CH/H, CH/F, CH/P, CH/Q
	CH	10	42.88	44.08	31.47	53.84	7.792	
	F	12	8.160	7.363	3.704	14.18	2.971	
	H	5	5.749	5.794	1.636	9.217	3.029	
	P	7	3.106	1.149	0.496	12.70	4.364	
	Q	5	2.360	2.604	1.004	3.902	1.209	
Ti	A	4	0.272	0.271	0.110	0.438	0.134	CH/Q, H/F, F/Q
	CH	10	0.878	0.601	0.421	2.110	0.628	
	F	12	0.990	0.994	0.601	1.413	0.267	
	H	5	0.208	0.123	0.107	0.424	0.140	
	P	7	0.481	0.274	0.110	1.898	0.647	
	Q	5	0.061	0.089	<LOQ	0.109	0.056	
Zn	A	4	30.56	28.72	24.44	40.37	6.885	H/A, H/Q, A/P, Q/P
	CH	10	48.25	49.69	40.54	54.63	4.560	
	F	12	52.94	49.69	44.34	74.53	10.09	
	H	5	87.22	80.26	65.90	124.7	23.67	
	P	7	85.56	91.46	45.10	129.6	26.89	
	Q	5	37.09	32.51	24.20	54.57	14.06	

**Table 4:** Results of Ca, Cu, Fe, K, Mg, Mn, P and Zn determination in the analysed samples: LV—labelled value (value placed on the product label) [mg/100 g]; DV—determined value (value obtained during analysis) [mg/100 g]; DV vs. LV—percentage of determined value versus labelled value [%].

No.	Ca			Cu			Fe			K		
	LV	DV	DV vs. LV	LV	DV	DV vs. LV	LV	DV	DV vs. LV	LV	DV	DV vs. LV
1	-	-	-	-	-	-	7.90	6.78	85.8	-	-	-
5	638	505	78.9	1.60	1.61	101	7.70	6.80	88.3	920	565	61.5 ↓
6	606	508	83.9	1.40	1.81	130	6.90	7.35	107	-	-	-
8	631	576	91.3	-	-	-	-	-	-	-	-	-
11	624	455	73.0	-	-	-	9.60	6.89	71.7	407	748	184 ↑
18	-	-	-	1.20	1.30	108	-	-	-	-	-	-
21	195	201	103	0.40	1.38	346 ↑	17.1	9.11	53.2 ↓	762	798	105
24	-	-	-	-	-	-	5.70	6.61	116	-	-	-
29	-	-	-	2.85	2.23	78.2	24.1	18.9	78.3	-	-	-
**No.**	**Mg**			**Mn**			**P**			**Zn**		
	**LV**	**DV**	**DV vs. LV**	**LV**	**DV**	**DV vs. LV**	**LV**	**DV**	**DV vs. LV**	**LV**	**DV**	**DV vs. LV**
1	257	244	95.1	-	-	-	557	531	95.3	-	-	-
5	368	276	75.0	4.70	7.26	155 ↑	777	575	74.0	5.60	5.04	90.1
6	306	284	92.9	-	-	-	785	670	85.3	4.40	5.26	120
7	-	-	-	10.9	7.41	68.0	735	680	92.5	-	-	-
8	335	286	85.4	-	-	-	-	-	-	-	-	-
9	329	302	91.8	-	-	-	826	728	88.1	-	-	-
11	335	330	98.4	-	-	-	860	821	95.4	-	-	-
18	392	307	78.4	-	-	-	-	-	-	-	-	-
21	291	285	97.8	1.20	3.00	250 ↑	722	596	82.6	7.80	4.65	59.7 ↓
24	390	300	76.8	-	-	-	-	-	-	4.30	5.10	119
29	967	664	68.7	15.6	13.6	87.1	2130	1700	79.8	-	-	-
32	-	-	-	-	-	-	-	-	-	8.40	6.96	82.9
40	197	126	63.9 ↓	-	-	-	457	320	70.0	3.10	2.42	78.1

↑—a value higher than permitted by regulations [34]; ↓—a value lower than permitted by regulations [34].

**Table 5:** The list of analysed samples, along with the abbreviations used.

Sample No.	Type	Abbreviation	Certificate
1	Amaranth grains	A	PL-EKO-04
2			-
3			-
4			PL-EKO-03
5	Chia seeds	CH	PL-EKO-01
6			-
7			-
8			-
9			PL-EKO-06
10			-
11			-
12			-
13			PL-EKO-01
14			PL-EKO-07
15	Flax seeds	F	-
16			-
17			PL-EKO-07
18			-
19			-
20			-
21			-
22			-
23			-
24			-
25			-
26			-
27	Hemp seeds	H	-
28			PL-EKO-07
29			PL-EKO-06
30			PL-EKO-07
31			-
32	Pumpkin seeds	P	-
33			-
34			-
35			PL-EKO-01
36			-
37			-
38			-
39	Quinoa grains	Q	PL-EKO-06
40			-
41			-
42			-
43			-

**Table 6:** Microwave digestion process conditions.

Stage No.	Time [min]	E [W]	P [bar]	T_1_ [°C]	T_2_ [°C]
1	15	1500	120	70	220
2	10	1500	120	220	220

**Table 7:** Operational parameters and instrument settings for ICP-OES measurements.

Instrument Parameter	Conditions
Generator power	1150 W
Carrier gas	Argon
Plasma gas flow rate	12 L/min
Auxiliary gas flow rate	0.5 L/min
Nebulizer gas flow rate	0.5 L/min
Nebulizer	Quartz
Torch	Quartz

**Table 8:** Limits of quantification (LOQ), limits of quantification in mg/kg of the sample, wavelengths [nm] and linear range for ICP-OES analysis.

Element	Wavelength	LOQ	LOQ	Linear Range
		µg/L	mg/kg	mg/L
Ag	328.068 ^a^	5	0.83	0.01–10.0
Al	369.152 ^a^	31	5.17	
Ba	455.403 ^r^	20	3.33	
Ca	393.366 ^r^	42	7.00	
Cd	214.438 ^a^	5	0.83	
Co	228.616 ^a^	5	0.83	
Cr	267.716 ^a^	31	5.17	
Cu	224.700 ^a^	7	1.17	
Fe	238.204 ^a^	90	15.0	
K	766.490 ^r^	99	16.5	
Mg	279.553 ^a^	47	7.83	
Mn	257.610 ^a^	4	0.67	
Mo	202.030 ^a^	6	1.00	
Ni	231.604 ^a^	10	1.67	
P	177.495 ^a^	44	7.33	
Pb	220.353 ^a^	11	1.83	
S	180.731 ^a^	88	14.7	
Sn	189.989 ^a^	28	4.67	
Sr	421.552 ^r^	8	1.33	
Ti	334.941 ^a^	2	0.33	
Zn	213.856 ^a^	37	6.17	
Yb *	328.937 ^a^	10	1.67	

^a^—axial view; ^r^—radial view; *—internal standard (Yb).

**Table 9:** Validation results obtained for certified reference materials.

Element	INCT-MPH-2 Certified	INCT-MPH-2 Obtained x_mean_ ± SD	Recovery	1568b Certified	1568b Obtained x_mean_ ± SD	Recovery
	**mg/kg**	**mg/kg**	**%**	**mg/kg**	**mg/kg**	**%**
Al	670 ± 111	681 ± 15	101.6	4.21 ± 0.34	4.35 ± 0.19	103.3
Ba	32.5 ± 2.5	31.8 ± 0.9	97.85	-	-	-
Ca	1.08 ± 0.07 (%)	1.01 ± 0.02 (%)	101.9	118.4 ± 3.1	123 ± 0.9	103.9
Cd	0.199 ± 0.023	0.190 ± 0.19	95.48	0.0224 ± 0.0013	0.020 ± 0.003	89.29
Co	0.21 ± 0.025	0.20 ± 0.02	95.24	*0.0177* ± *0.0005*	0.016 ± 0.001	90.40
Cr	1.69 ± 0.13	1.61 ± 0.09	95.27	-	-	-
Cu	7.77 ± 0.53	7.75 ± 0.40	99.74	2.35 ± 0.16	2.38 ± 0.09	101.3
Fe	*460*	499	108.5	7.42 ± 0.44	7.45 ±0.12	100.4
K	1.91 ± 0.12 (%)	1.95 ± 0.10 (%)	102.1	1282 ± 11	1292 ± 1.0	100.8
Mg	0.29 ± 0.02 (%)	0.32 ± 0.01 (%)	110.3	559 ± 10	555 ± 8	99.28
Mn	191 ± 12	191 ± 10	100.0	19.2 ± 1.8	18.9 ± 1.1	98.44
Mo	*0.52*	0.49 ± 0.01	94.23	1.451 ± 0.048	1.350 ± 0.082	93.04
Ni	1.57 ± 0.16	1.42 ± 0.05	90.45	-	-	-
P	*0.25* (%)	0.26 ± 0.01 (%)	104.0	1530 ± 40	1560 ± 11	102.0
Pb	2.16 ± 0.23	2.12 ± 0.11	98.15	*0.008* ± *0.003*	0.007 ± 0.002	87.50
S	0.24 ± 0.01 (%)	0.25 ± 0.01 (%)	104.2	1200 ± 10	1208 ± 3.2	100.7
Sr	37.6 ± 2.7	38.1 ± 0.9	101.3	-	-	-
Ti	*34*	31.5± 0.8	92.65	-	-	-
Zn	33.5 ± 2.1	34.5 ± 0.2	103.0	19.42 ± 0.26	19.56 ± 0.19	100.7

italic value—information value.

## Data Availability

The raw data supporting the conclusions of this article will be made available by the authors on request.
